# *Rhizobium rhizogenes*-mediated hairy-root transformation of daughter plants from the model strawberry *Fragaria vesca*’s stolons

**DOI:** 10.5511/plantbiotechnology.24.0925a

**Published:** 2024-12-25

**Authors:** Shigeru Hanano, Koichiro Otake, Shusei Sato

**Affiliations:** 1 Graduate School of Life Sciences, Tohoku University, Sendai, Miyagi 980-8577, Japan; 2 Kazusa DNA Research Institute, Chiba 292-0818, Japan

**Keywords:** genetic modification, hairy-root transformation, *Rhizobium rhizogenes*, stolon, strawberry

## Abstract

Strawberry, a member of the *Fragaria* genus within the Rosaceae family, is one of the most cherished fruits worldwide. This perennial herbaceous plant also serves as a model for studying the Rosaceae family. Despite the complex polyploidy of strawberries, extensive efforts in traditional breeding over the years have resulted in improvements in yield, fruit size and shape, berry quality, and various other aspects of strawberry production. However, in addition to these conventional methods, advanced genetic technologies such as genetic modification and gene editing in intricate polyploidy varieties of strawberry are also required. Here, we present the *Rhizobium rhizogenes*-mediated hairy-root transformation of daughter plants from the model strawberry *Fragaria vesca*’s stolons (also called runners), which exhibit diploid genomes. As a case study, new daughter plants were cut from the stolons, infected with *R. rhizogenes* harboring the *mVENUS* gene under Cauliflower mosaic virus 35S promoter, and then transferred on vermiculite-filled pots. After a couple of months of growth, fluorescence was observed in a few adventurous roots of the daughter plants. The hairy root transformation of daughter plants isolated from its vegetative propagation circumvents the need for seed production or callus formation and subsequent plant regeneration, which are often problematic for maintaining preferred genetic traits in complex ploidy levels. This method, which excludes genetic modification of the above-ground parts, especially the edible fruits, will open new avenues for strawberry breeding, particularly in the areas of plant nutrient absorption and fostering growth through interactions with microorganisms.

Strawberries are globally cultivated for their adaptability and economic importance (Debnath and Teixeira Da Silva 2007). They belong to the Fragaria genus in the Rosaceae family and are rich in valuable metabolites such as essential nutrients and antioxidants beyond their appearance and taste (Dias et al. 2016). Furthermore, the strawberry is one of the model perennial herbaceous plants for studying the Rosaceae family due to its rapid growth, small genome, short reproductive cycle, and facile vegetative and generative propagation (Shulaev et al. 2011). There are three main ways to propagate strawberry plants: division and transplantation of their crowns, germination from seeds, or harnessing stolons, also called runners, for efficient clonal reproduction, which is particularly crucial in managing the intricate ploidy levels, observed in strawberry varieties (e.g. 2n=8x=56 in *Fragaria*×*ananassa*) (Hirakawa et al. 2014). Conventional genetic breeding via classical fertilization has improved various strawberry traits such as yield, fruit size and shape, and berry quality similar to achievements in other plant species (Isobe et al. 2013; Yamamoto et al. 2021). However, genetic breeding in cultivated strawberry is labor-intensive and time-consuming due to complex ploidy levels, underlining the significance of exploring efficient genetic modification strategies during clonal propagation.

Recent advances in genetic transformation via *Rhizobium radiobactor* (formerly known as *Agrobacterium tumefaciens*) have significantly improved the strawberry genetic traits, enhancing resistance to virus, fungi, insects, herbicides, and stress, as well as achieving better quality (Nehra et al. 1990; Pantazis et al. 2013; Qin et al. 2008; Ricardo et al. 2003). In general, *R. radiobactor*-mediated transformation produces stable and targeted transformants as an efficient tool. However, *R. radiobactor*-mediated transformation, which involves callus formation and regeneration, faces challenges related to the efficiency of regeneration and subsequent organogenesis, as well as the selection and recovery of transformed cells with antibiotic resistance. Despite the occurrence of genome rearrangements and somatic mutations during callus formation and regeneration, addressing these mutations through backcrossing is not realistic in polyploid strawberries, since plants grown from seeds may exhibit different traits from the parental plants that produced the seeds. Moreover, the stable introduction of foreign genes throughout the entire plant body, including fruits, often faces criticism due to concerns about the presence of transgenes in the edible parts.

In contrast to *R. radiobactor*, *Rhizobium rhizogenes* (formerly known as *Agrobacterium rhizogenes*) introduces foreign genes only into adventitious roots induced from plant wounds (Daspute et al. 2019; Nilsson and Olsson 1997; Tatsumi et al. 2020; Tepfer 1984; Toyoda et al. 1991; Yazaki et al. 1998). *R. rhizogenes*-mediated hairy-root transformation in strawberries has been reported in leaf discs and aboveground sections with cotyledons and hypocotyls from seedlings over the past 20 years (Kyo and Shirai 1990; Toyoda et al. 1993; Yan et al. 2023). While the hairy-root transformation is suitable for transient assays in roots grown from seedlings, challenges remain in maintaining genetic traits and obtaining stable transformants. Here, we report a new method of *R. rhizogenes*-mediated hairy-root transformation of daughter plants growing on strawberry stolons (Figures 1 and 2). This method does not require callus formation and plant regeneration, transforms only the hairy roots without inserting foreign genes into the above-ground parts, and allows for the expression of foreign genes without seed propagation (Figure 1B).

**Figure 1:**
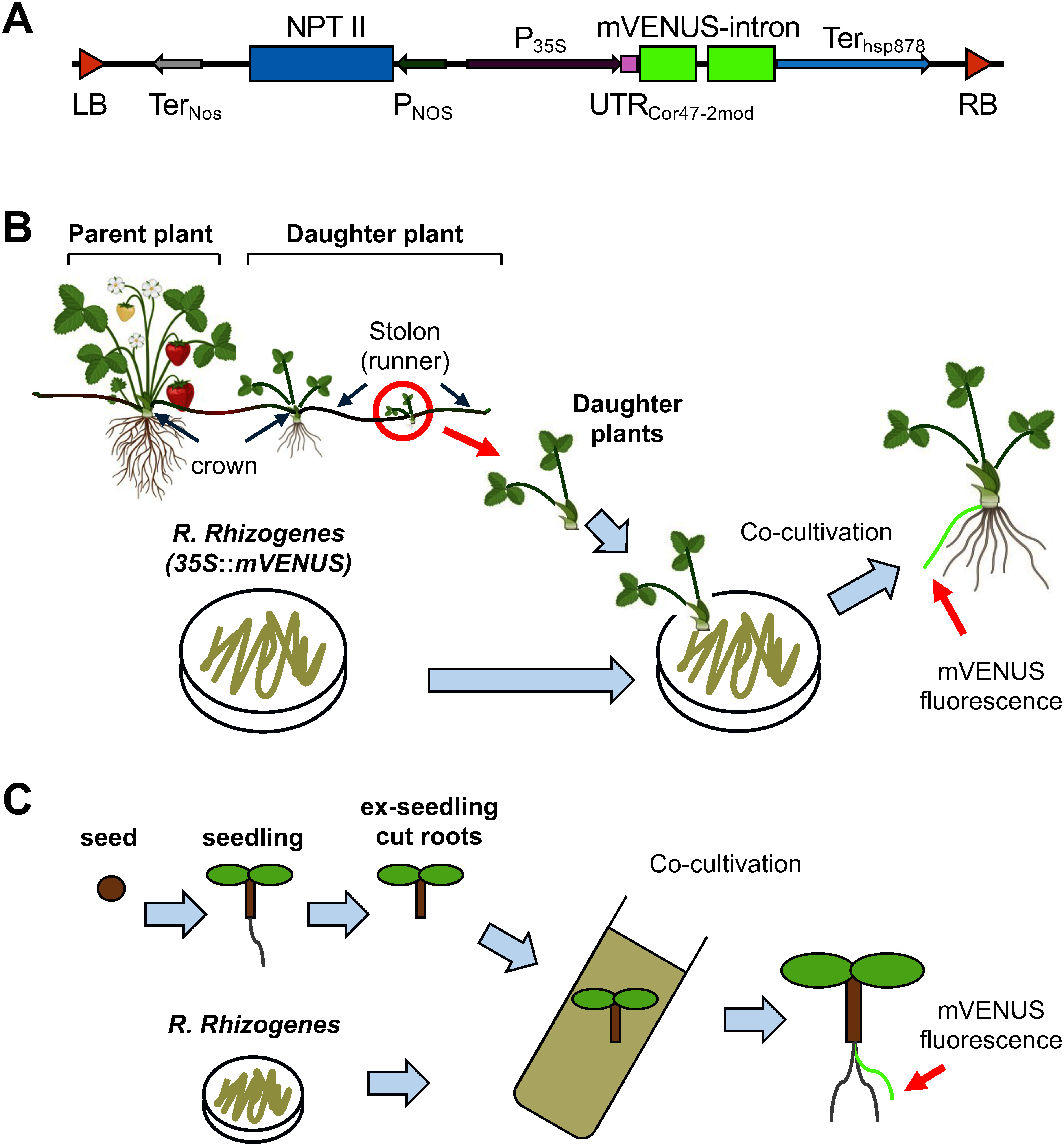
Figure 1. Schematic representation of the *35S*::*mVENUS* construct and transformation of daughter plant grown on stolon. (A) The *35S*::*mVENUS* construct used in this study. LB, left border; RB, right border; NPT II, neomycin phosphotransferase Type II gene; NOS, nopaline synthase; 35S, Cauliflower mosic virus 35S; P, promoter; Ter, terminator; UTR_cor47-2mod_, 5′-untranslated region for a highly efficient translation; hsp878, heat shock protein 878. (B) Schematic representation of the transformation process for daughter plants grown on stolons. Daughter plants harvested from stolons elongated from parent plants were infected and co-cultivated with *Rhizobium rhizogenes* harboring foreign genes. Some of the emerging roots from daughter plants could contain foreign genes. (C) Schematic representation of seedling transformation. Ex-seedlings with cut roots were co-cultivated with *R. rhizogenes*.

**Figure 2:**
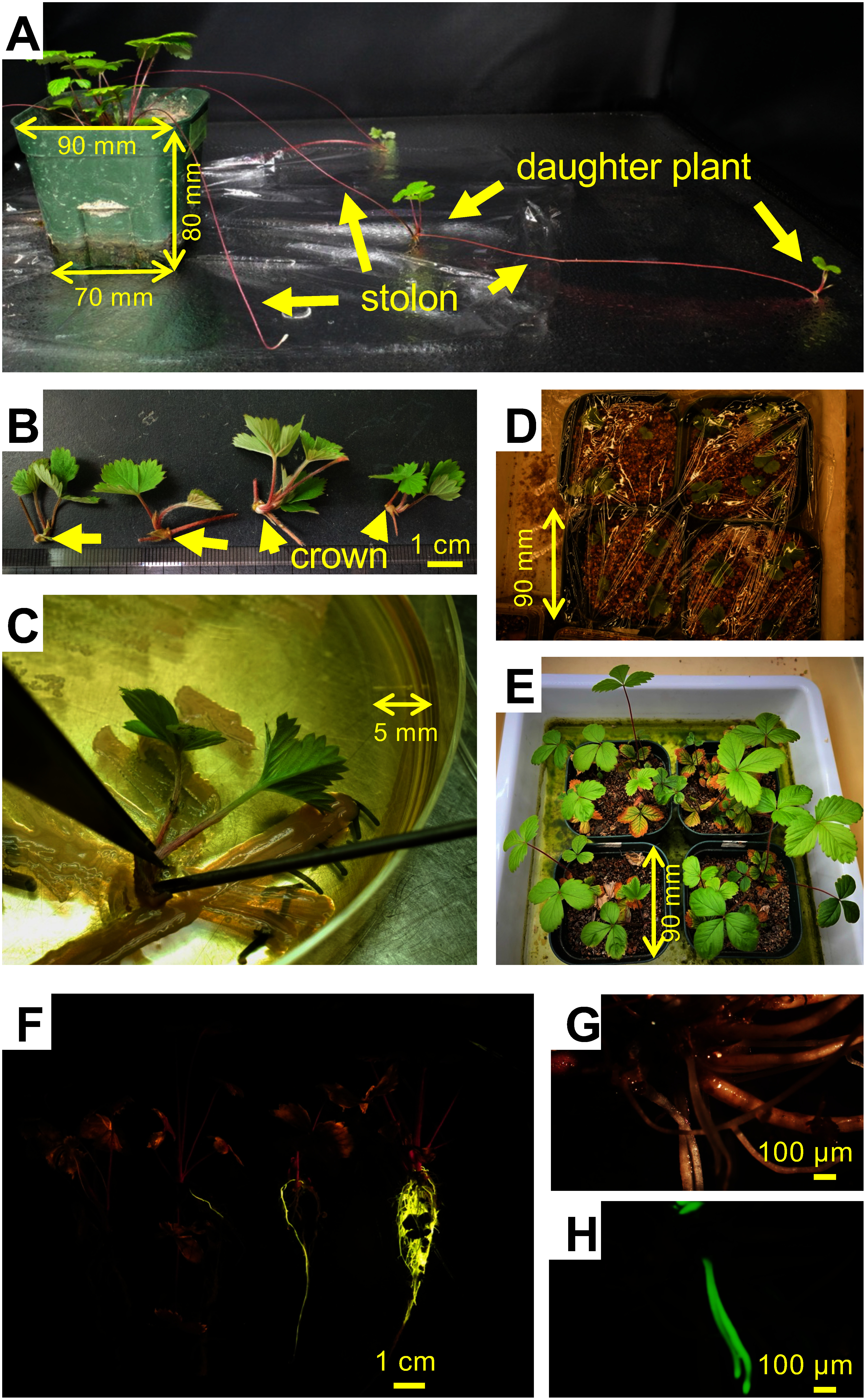
Figure 2. Daughter plant transformation reveals transgenic roots. (A) Daughter plants growing on strawberry *Fragaria vesca* stolons. The dimensions of the pot—7 cm×7 cm at base, 9 cm×9 cm at the top, and 8 cm in depth—were represented to estimate the size of plants. (B) Daughter plants were cut from the stolons of the parental plants, (C) placed on *R. rhizogenes* colonies harboring the *35S*::*mVENUS* plasmid and infected with bacteria by puncturing their crowns with a syringe on a LB plates. The mark on the Petri dish indicated was 5 mm. (D) Infected daughter plants were planted in vermiculite-filled pots with a wrap. (E) The wrap was removed after 1 weeks, and daughter plants were grown on vermiculites for 1 to 2 months. A top edge length of each pot was 9 cm. (F) Variation in mVENUS expression in the transgenic roots of the strawberry *F. vesca*. mVENUS fluorescence photographs were captured with a PowerShot G16 digital camera (Canon, Tokyo) with a PA-V300f filter under 475 to 515 nm LED (Bio Craft, Tokyo). Microscopy images were observed under a SZX12 fluorescence microscope system (G) without and (H) with a GFP filter (Olympus, Tokyo). The difference in fluorescence colors, (F) yellow and (H) green, depends on the filter used.

We chose the diploid woodland strawberry, *Fragaria vesca* Hawaii-4 (2n=14), which is well-characterized with a small genome, short reproductive cycle, and both vegetative and generative propagation (Shulaev et al. 2011), as a model for studying the hairy-root transformation of daughter plants from strawberry stolons. Details about the experimental protocols are described in Supplementary Methods. Surface-sterilized *F. vesca* seeds were aseptically grown on 1/2 Gamborg’s B5 medium 0.9% agar plates without sugar and vitamin. Four- to six-week-old seedlings were transferred and grown on vermiculite-filled pots in the greenhouse with a heating system and maintained by propagating daughter plants on stolons. For daughter plants transformation, stolons with daughter plants sized 2 to 4 cm were harvested (Figure 2A, B).

To evaluate transgenic efficiencies for the hairy-root transformation, *R. rhizogenes* A13 strain (MAFF-210266)(Chen et al. 1995) harboring a pRI909 vector containing *mVENUS*-intron driven by the CaMV 35S promoter with the hsp878 terminator (*35S*::*mVENUS*) (Matsui et al. 2014; Otake et al. 2023; Yamasaki et al. 2018) was utilized (Figure 1A). The *35S*::*mVENUS* plasmid was introduced into *R. rhizogenes* by electroporation (Wen-Jun and Forde 1989). The *R. rhizogenes* strain was grown in a 28°C dark growth chamber on LB plates for 3 days.

Daughter plants sized 2–4 cm were cut from the stolons of their parent plants, placed on *R. rhizogenes* colonies harboring the *35S*::*mVENUS* vector, and infected with the bacteria by puncturing the crown of the daughter plant with a syringe on an LB plate (Figure 2C, Supplementary Methods). The infected daughter plants were transplanted into vermiculite-filled pots and wrapped to prevent drying (Figure 2D). After one week, the wrap was removed, and the infected daughter plants were grown in vermiculite for 1 to 2 months till adventitious roots emerged (Figure 2E).

To compare the ratio of transgenic efficiency, we also performed the hairy-root transformation of seedlings as described previously (Díaz et al. 2005) (Figure 1C, Supplementary Methods). One- to two-week-old etiolated seedlings, aseptically grown on 1/2 B5 agar plates and cut at the lower part of the hypocotyl, were co-incubated with *R. rhizogenes* harboring the *35S*::*mVENUS* on 1/2 B5 agar plates without antibiotics for 3 to 4 days. Subsequently, they were transferred and vertically grown on 1/2 B5 agar plates containing 12.5 µg l^−1^ of meropenem for 2 to 4 weeks to generate hairy roots.

In transformation of daughter plants from stolons, we successfully obtained transgenic plants with adventitious roots exhibiting strong *mVENUS* fluorescence 1 to 2 months post-infection (Figure 2F, G, H). The frequencies of the plants exhibiting *mVENUS*-positive roots ranged from 25% to 53.3% of the total plants used in the experiments (Table 1). The number of transformed hairy roots per plant exhibiting fluorescence ranged from one to several (Supplementary Methods, Figure S1). The transformation efficiency of daughter plants was comparable to that of seedlings, which were used as a control; the transgenic efficiencies in the cut-off roots of seedlings ranged from 10% to 73.7% of the total inoculated plants (Table 1). *mVENUS* expression was observed in several emerging roots from the cut end 3 weeks after infection. Thus, *R. rhizogenes* is suitable for the hairy-root transformation of daughter plants grown on the strawberry stolons as well as seedlings cut at their hypocotyls.

**Table 1:** Table 1. The ratio of survival and transgenic efficiencies.

	Type of plants	Total	Survive	*mVENUS*	No signal	Transgenic rate per total (%)
Exp. 1	Daughter plants	12	8	3	5	25.0
Exp. 2	Daughter plants	15	13	8	5	53.3
Exp. 3	Daughter plants	15	9	5	4	33.3
Exp. 4	Daughter plants	16	14	7	7	43.8
Exp. 5	Daughter plants	16	15	6	9	37.5
Exp. S1	Seedlings	19	19	14	5	73.7
Exp. S2	Seedlings	37	35	10	25	27.0
Exp. S3	Seedlings	22	20	6	14	27.3
Exp. S4	Seedlings	20	10	2	8	10.0
Exp. S5	Seedlings	45	41	10	31	22.2
Exp. S6	Seedlings	17	17	4	13	23.5

Survival and transgenic efficiencies in a series of experiments on daughter plants from stolons (Exp. 1 to 5) and seedlings cut at the lower part of hypocotyl (Exp. S1 to S6). Several plants did not survive due to drought or bacterial and fungal infections. The transgenic rate (%) is calculated as the number of plants expressing mVENUS fluorescence divided by the total number of plants used in the experiments.

Here, we present a novel method for the *R. rhizogenes*-mediated hairy-root transformation of daughter plants grown on strawberry stolons, aiming to address the breeding challenges in the economically significant fruit crop. By harnessing the unique physiology of stolons, we developed a protocol that introduces foreign genes into progeny generated through vegetative reproduction. This approach bypasses the conventional limitations associated with seed propagation and tissue-culture-based transformation methods in higher polyploid plants. Unlike *R. rhisozogenes*-mediated transformation of seedlings (Figure 1C), that of daughter plants from the stolons avoids seed propagation, thus preserving the parental genetic traits.

Our protocol involves cutting daughter plants from the stolons and infecting their crowns, which contain meristems, with the *R. rhizogenes* A13 strain. One to two months post-infection, we observed *mVENUS* expression in some roots emerging from the cut-out daughter plants. Significantly, these daughter plants with transgenic roots continue to grow as clones of their parental plants during the vegetative reproduction stage, thus avoiding seed propagation, callus formation, and plant regeneration. In higher-order polyploid plants, where genetic traits segregate across homologous chromosomes, vegetative propagation without seed is especially important. This suggests that the daughter progenies with transgenic roots maintain the same genetic traits as their parent plants. Extending the *R. rhizogenes*-mediated hairy-root transformation to other strawberry varieties and plant species could allow the expression of foreign genes only in the roots, maintaining the genotype in higher-order ploidy plants and preserving non-genetically modified edible parts.

*R. rhizogenes* induces adventitious roots with transgenes without requiring callus formation and regeneration. This hairy-root transformation is suitable for transient expression analysis of foreign genes, allowing the study of promoters and gene functions directly in roots, as transgenic roots are visible in their T0 generation (Yan et al. 2023). Earlier studies in plant biotechnology have demonstrated that *R. rhizogenes*-mediated hairy-root transformation can produce secondary metabolites in medicinal plants (Motomori et al. 1995; Yazaki et al. 1998). While *R. radiobactor* is commonly used in planta assays in mostly dicots and some monocots (Pantazis et al. 2013), it is often inefficient and time-consuming due to the necessity for callus formation and regeneration. In contrast, *R. rhizogenes* can infect a broader range of hosts, making its transformation applicable to more plant species. The *R. rhizogenes*-mediated hairy-root transformation, where genetic modification occurs only in the roots, may improve nutrient absorption, abiotic stress tolerance, and disease resistance in plant roots. Furthermore, combining this transformation system with RNA or protein transport mechanisms from roots to shoots might enable the modification of above-ground phenotypes (Zhang et al. 2016).

Regenerating stable transformants after the *R. rhizogenes*-mediated hairy-root transformation remains challenging. However, transformed roots offer an opportunity to generate transgenic plants by inducing callus formation and subsequent plant regeneration through the application of phytohormones (Yan et al. 2023). Recent advances in plant development have identified several candidates that induce shoots from roots (Debernardi et al. 2020; Hanano et al. 2020; Ikeuchi et al. 2013; Kyo et al. 2018; Yarra and Krysan 2023). These advancements may enable the conversion of *R. rhizogenes*-mediated transgenic roots into shoots or other organs, ultimately leading to the development of intact transgenic plants. Combining *R. rhizogenes*-mediated transformation with shoot-inducible genes holds significant potential for advancing plant science and biotechnology.

## Abbreviations

CaMV: Califlower mosic virus
cor47: cold-regulated 47 gene 5′-untranslated region
*F. vesca*: *Fragaria vesca*
hsp: heat shock protein
LB medium: Luria–Bertani medium
1/2 B5 agar plate: 1/2 Gamborg’s B5 medium 0.9% agar plate
*R. radiobactor*: *Rhizobium radiobactor* (former name is *Agrobacterium tumefaciens*)
*R. rhizogenes*: *Rhizobium rhizogenes* (former name, *Agrobacterium rhizogenes*)

## Description of Supplementary Files

Supplementary Methods. Seed sterilization and growth. Transformation of *Rhizobium rhizogenes* by electroporation. Transformation of daughter plants from strawberry stolons. Hairy-root transformation of strawberry seedlings.

Supplementary Figure S1. The number of transformed hairy roots in each plant.

## References

[RChen1995] Chen L-H, Hata T, Yamakawa Y, Suzuki Y (1995) The effects of preservation temperatures and periods on hairy roots inducing ability of *Agrobacterium rhizogenes.* *Plant Tissue Cult Lett* 12: 94–96

[RDaspute2019] Daspute AA, Yunxuan X, Gu M, Kobayashi Y, Wagh S, Panche A, Koyama H (2019) *Agrobacterium rhizogenes*-mediated hairy roots transformation as a tool for exploring aluminum-responsive genes function. *Future Sci OA* 5: FSO364 30906565 10.4155/fsoa-2018-0065 PMC6426172

[RDebernardi2020] Debernardi JM, Tricoli DM, Ercoli MF, Hayta S, Ronald P, Palatnik JF, Dubcovsky J (2020) A GRF–GIF chimeric protein improves the regeneration efficiency of transgenic plants. *Nat Biotechnol* 38: 1274–1279 33046875 10.1038/s41587-020-0703-0 PMC7642171

[RDebnath2007] Debnath SC, Teixeira Da Silva JA (2007) Strawberry culture in vitro: applications in genetic transformation and biotechnology. *Fruit Veg Cereal Sci Biotechnol* 1: 1–12

[RDias2016] Dias MI, Barros L, Morales P, Cámara M, Alves MJ, Oliveira MB, Santos-Buelga C, Ferreira IC (2016) Wild: *Fragaria vesca* L. fruits: A rich source of bioactive phytochemicals. *Food Funct* 7: 4523–4532 27775146 10.1039/c6fo01042c

[d67e862] Díaz CL, Gronlund M, Schlaman HRM, Spaink HP (2005) Induction of hairy roots for symbiotic gene expression studies. In: *Márquez AJ (ed) Lotus Japonicus Handbook*. Springer, Netherlands, pp 261–277

[RHanano2020] Hanano S, Tomatsu H, Ohnishi A, Kobayashi K, Kondo Y, Betsuyaku S, Takita E, Ogata Y, Ozawa K, Suda K, et al. (2020) An artificial conversion of roots into organs with shoot stem characteristics by inducing two transcription factors. *iScience* 23: 101332 32668199 10.1016/j.isci.2020.101332 PMC7385925

[RHirakawa2014] Hirakawa H, Shirasawa K, Kosugi S, Tashiro K, Nakayama S, Yamada M, Kohara M, Watanabe A, Kishida Y, Fujishiro T, et al. (2014) Dissection of the octoploid strawberry genome by deep sequencing of the genomes of fragaria species. *DNA Res* 21: 169–181 24282021 10.1093/dnares/dst049 PMC3989489

[RIkeuchi2013] Ikeuchi M, Sugimoto K, Iwase A (2013) Plant callus: Mechanisms of induction and repression. *Plant Cell* 25: 3159–3173 24076977 10.1105/tpc.113.116053 PMC3809525

[RIsobe2013] Isobe SN, Hirakawa H, Sato S, Maeda F, Ishikawa M, Mori T, Yamamoto Y, Shirasawa K, Kimura M, Fukami M, et al. (2013) Construction of an integrated high density simple sequence repeat linkage map in cultivated strawberry (*Fragaria*×*ananassa*) and its applicability. *DNA Res* 20: 79–92 23248204 10.1093/dnares/dss035 PMC3576660

[RKyo2018] Kyo M, Maida K, Nishioka Y, Matsui K (2018) Coexpression of WUSCHEL related homeobox (WOX) 2 with WOX8 or WOX9 promotes regeneration from leaf segments and free cells in *Nicotiana Tabacum* L. *Plant Biotechnol (Tokyo)* 35: 23–30 31275034 10.5511/plantbiotechnology.18.0126a PMC6543738

[RKyo1990] Kyo M, Shirai H (1990) Application of Agrobacterium-mediated gene transfer method in Fragaria. *Tech Bull Fac Agr Kagawa Univ* 42: 205–211

[RMatsui2014] Matsui T, Sawada K, Takita E, Kato K (2014) The longer version of *Arabidopsis thaliana* heat shock protein 18.2 gene terminator contributes to higher expression of stably integrated transgenes in cultured tobacco cells. *Plant Biotechnol (Tokyo)* 31: 191–194

[RMotomori1995] Motomori Y, Shimomura K, Mori K, Kunitake H, Nakashima T, Tanaka M, Miyazaki S, Ishimaru K (1995) Polyphenol production in hairy root cultures of *Fragaria*×*ananassa.* *Phytochemistry* 40: 1425–1428

[RNehra1990] Nehra NS, Chibbar RN, Kartha KK, Datla RS, Crosby WL, Stushnoff C (1990) Genetic transformation of strawberry by *Agrobacterium tumefaciens* using a leaf disk regeneration system. *Plant Cell Rep* 9: 293–298 24226936 10.1007/BF00232854

[RNilsson1997] Nilsson O, Olsson O (1997) Getting to the root: The role of the *Agrobacterium rhizogenes rol* genes in the formation of hairy roots. *Physiol Plant* 100: 463–473

[ROtake2023] Otake K, Kugou K, Robertlee J, Ohzeki JI, Okazaki K, Hanano S, Takahashi S, Shibata D, Masumoto H (2023) *De novo* induction of a DNA–histone H3K9 methylation loop on synthetic human repetitive DNA in cultured tobacco cells. *Plant J* 114: 668–682 36825961 10.1111/tpj.16164

[RPantazis2013] Pantazis CJ, Fisk S, Mills K, Flinn BS, Shulaev V, Veilleux RE, Dan Y (2013) Development of an efficient transformation method by *Agrobacterium tumefaciens* and high throughput spray assay to identify transgenic plants for woodland strawberry (*Fragaria vesca*) using NPTII selection. *Plant Cell Rep* 32: 329–337 23160638 10.1007/s00299-012-1366-1

[RQin2008] Qin Y, Teixeira da Silva JA, Zhang L, Zhang S (2008) Transgenic strawberry: State of the art for improved traits. *Biotechnol Adv* 26: 219–232 18280082 10.1016/j.biotechadv.2007.12.004

[RRicardo2003] Ricardo VG, Coll Y, Castagnaro A, Diaz Ricci JC (2003) Transformation of a strawberry cultivar using a modified regeneration medium. *HortScience* 38: 277–280

[RShulaev2011] Shulaev V, Sargent DJ, Crowhurst RN, Mockler TC, Folkerts O, Delcher AL, Jaiswal P, Mockaitis K, Liston A, Mane SP, et al. (2011) The genome of woodland strawberry (*Fragaria vesca*). *Nat Genet* 43: 109–116 21186353 10.1038/ng.740 PMC3326587

[RTatsumi2020] Tatsumi K, Ichino T, Onishi N, Shimomura K, Yazaki K (2020) Highly efficient method of *Lithospermum erythrorhizon* transformation using domestic *Rhizobium rhizogenes* strain A13. *Plant Biotechnol (Tokyo)* 37: 39–46 32362747 10.5511/plantbiotechnology.19.1212a PMC7193830

[RTepfer1984] Tepfer D (1984) Transformation of several species of higher plants by *Agrobacterium rhizogenes*: Sexual transmission of the transformed genotype and phenotype. *Cell* 37: 959–967 6744417 10.1016/0092-8674(84)90430-6

[RToyoda1991] Toyoda H, Hosoi Y, Yamamoto A, Nishiguchi T, Maeda K, Takebayashi T, Shiomi T, Ouchi S (1991) Transformation of melon (*Cucumis melo* L.) with *Agrobacterium rhizogenes.* *Plant Tissue Cult Lett* 8: 21–27

[RToyoda1993] Toyoda H, Kami C, Sumitani K, Zheng SJ, Hosoi Y, Ouchi S (1993) Transformation of Japanese cultivars of strawberry with *Agrobacterium rhizogenes.* *Plant Tissue Cult (Syokubutsu Soshiki Baiyo)* 10: 92–94 (in Japanese)

[RWen1989] Wen-Jun S, Forde BG (1989) Efficient transformation of Agrobacterium spp. by high voltage electroporation. *Nucleic Acids Res* 17: 8385 2682529 10.1093/nar/17.20.8385 PMC334991

[RYamamoto2021] Yamamoto E, Kataoka S, Shirasawa K, Noguchi Y, Isobe S (2021) Genomic selection for F1 hybrid breeding in strawberry (*Fragaria*×*ananassa*). *Front Plant Sci* 12: 645111 33747025 10.3389/fpls.2021.645111 PMC7969887

[RYamasaki2018] Yamasaki S, Sanada Y, Imase R, Matsuura H, Ueno D, Demura T, Kato K (2018) *Arabidopsis thaliana* cold-regulated 47 gene 5′-untranslated region enables stable high-level expression of transgenes. *J Biosci Bioeng* 125: 124–130 28918993 10.1016/j.jbiosc.2017.08.007

[RYan2023] Yan H, Ma D, Yi P, Sun G, Chen X, Yi Y, Huang X (2023) Highly efficient *Agrobacterium rhizogenes*-mediated transformation for functional analysis in woodland strawberry. *Plant Methods* 19: 99 37742022 10.1186/s13007-023-01078-y PMC10517450

[RYarra2023] Yarra R, Krysan PJ (2023) GRF-GIF duo and GRF-GIF-BBM: Novel transformation methodologies for enhancing regeneration efficiency of genome-edited recalcitrant crops. *Planta* 257: 60 36801980 10.1007/s00425-023-04096-1

[RYazaki1998] Yazaki K, Tanaka S, Matsuoka H, Sato F (1998) Stable transformation of *Lithospermum erythrorhizon* by *Agrobacterium rhizogenes* and shikonin production of the transformants. *Plant Cell Rep* 18: 214–219 30744223 10.1007/s002990050559

[RZhang2016] Zhang W, Thieme CJ, Kollwig G, Apelt F, Yang L, Winter N, Andresen N, Walther D, Kragler F (2016) tRNA-related sequences trigger systemic mRNA transport in plants. *Plant Cell* 28: 1237–1249 27268430 10.1105/tpc.15.01056 PMC4944404

