# Supplementary material for: Rhizobium rhizogenes-mediated hairy-root transformation of daughter plants from the model strawberry Fragaria vesca’s stolons
Source: Plant Biotechnol (Tokyo). 2024 Dec 25;41(4):453–8. doi: 10.5511/plantbiotechnology.24.0925a (PMC11897731; doi:10.5511/plantbiotechnology.24.0925a)
Supplement: Supplementary Data [file plantbiotechnology-41-4-24.0925a-s001.pdf]

Supplementary Methods

Rhizobium rhizogenes -mediated Hairy-root Transformation of Daughter Plants from the Model Strawberry *Fragaria vesca*'s Stolons

Authors

Shigeru Hanano[1], Koichiro Otake[2] and Shusei Sato[1]

[1]Graduate School of Life Sciences, Tohoku University, Sendai 980-8577, Japan

[2]Kazusa DNA Research Institute, Chiba 292-0818, Japan

**Seed Sterilization and Growth**	p. 2
**Transformation of *Rhizobium rhizogenes* by Electroporation**	P. 4
**Transformation of Daughter Plants from Strawberry Stolons**	P. 6
**Hairy-root Transformation of Strawberry Seedlings**	P. 7
**References**	P. 9
**The number of transformed hairy roots in each plant**	P. 10

Seed Sterilization and Growth

Materials

1. Strawberry seeds. We used diploid woodland strawberry, *Fragaria vesca* Hawaii-4 (2n = 14).
2. 15 mL Falcon tube
3. 70% ethanol
4. 2% sodium hypochlorite solution with 0.1% tween-20
5. Sterile water
6. 0.1% agar
7. 1/2 Gamborg's B5 medium 0.9% agar plates (Gamborg et al., 1968)(FUJIFILM-WAKO, Japan). Note: We did not add sugar and vitamins to the medium to prevent bacterial overgrowth.

Methods

1. Put ~1 mL vol. of *F. vesca* seeds in 15 mL Falcon tube.
2. Add 5 mL of 70% ethanol to the tube and mix gently by inverting for 3 min to rinse the seeds.
3. Spin down by centrifugation at 500 x g for 5 seconds.
4. Discard the supernatant ethanol.
5. Add 5 mL of 2% sodium hypochlorite solution with 0.1% tween-20 and rinse the seeds gently by inverting for 15 minutes for surface sterilization. Recommendation: change the sodium hypochlorite solution during sterilization, e.g. repeat three times for 5-minute sterilizations.
6. Spin down by centrifugation at 500 x g for 5 seconds.
7. Discard the supernatant.
8. Wash the seeds by adding 5 mL of sterile water, mix gently by inverting several times, spin down by centrifugation, and discard the supernatant. Repeat this washing process three times.
9. Add 5 mL of 0.1% agar and aseptically sow the seeds on two of 1/2 Gamborg's B5 medium 0.9% agar plates.
10. Keep the plates at 4ºC for 2 to 3 days under dark conditions for imbibition.

If you store the seeds at 4 ºC for more than one month, this step is optional.

11. Move the plates to an appropriate growth condition. In our experiments, seedlings were grown at 22ºC under 16-hour light/8-hour dark cycles with 80 µmol m^{-2} sec^{-1} cool white-fluorescent light for 1 to 2 weeks till they germinated. For hairy-root

transformation of seedlings, the germinated seedlings were used.

12. Transplant four- to six-week-old seedlings into pots filled with vermiculite and grow them in a greenhouse with a heating system.

13. Maintain strawberry plants by propagating daughter plants on stolons.

14. For daughter plants transformation, harvest stolons with 2 to 4 cm-sized daughter plants.

Transformation of *Rhizobium rhizogenes* by Electroporation

Materials

1. *Rhizobium rhizogenes* A13 strain (MAFF-210266)
2. YEB medium (LB medium is also acceptable)
3. Sterilized water
4. Sterilized 10% glycerol
5. Sterilized 1.5 mL Eppendorf tubes
6. YEB 0.9% agar plate in sterile petri dish (ø9 cm) (LB is also acceptable)
7. A binary plasmid DNA, e. g. pRI909 vector that contains *mVENUS*-intron driven by CaMV 35S promoter with hsp878 terminator (*35S*::*mVENUS*) (Matsui et al., 2014; Yamasaki et al., 2018; Otake et al., 2023)
8. 0.1 cm electroporation cuvette (Bio-Rad, USA)
9. Bio-Rad Gene-Pulser electroporation system (Bio-Rad, USA)

Methods

Preparation of Competent Cells

1. Pick up a couple of *R. rhizogenes* colonies and inoculate them in 5 mL YEB liquid medium with rifampicin and streptomycin. Culture the bacteria by shaking at 100 rpm at 28ºC for overnight.
2. Transfer 500 µL of the overnight culture into 25 mL YEB liquid medium with rifampicin and streptomycin. Culture the bacteria by shaking at 100 rpm at 28ºC for 5 to 6 hours until OD_{600} reaches 0.15 to 0.2.
3. Cool culture cells on ice for 10 minutes.
4. Centrifuge the cells at 4000 x g for 10 minutes at 4ºC and discard the supernatant.
5. Wash the cells by adding 10 mL of pre-cooled sterile water, mix gently by inverting several times, centrifuge, and discard the supernatants. Repeat this washing process three times. Caution: Remove YEB medium containing salts by washing to avoid the risk for electrical shocks during electroporation.
6. Wash the cells by adding 5 mL of pre-cooled 10% glycerol.
7. Centrifuge the cells at 4000 x g for 10 minutes at 4ºC and discard the supernatant.
8. Add 1 mL of pre-cooled 10% glycerol and resuspend the pellet.
9. Dispense 40 µL aliquots into 1.5 mL Eppendorf tubes, freeze in liquid nitrogen, and store at -70°C.

Transformation of R. rhizogenes by Electroporation

1. Pick out the tube containing *R. rhizogenes* competent cells from a -70ºC freezer and place it on ice to thaw.
2. Add 1-5 µL of plasmid DNA solution (100 ng to 1 µg) to 40 µL of cells, and mix gently by pipetting.
3. Introduce the plasmid DNA into the cells using a Bio-Rad Gene-Pulser electroporation system (Bio-Rad, USA) with the following settings: field strength of 1.25 kV, capacitance of 25 µF, and resistance of 200 ohms, in a pre-cooled 0.1 cm electroporation cuvette (Wen-Jun & Forde, 1989). The time constant should be approximately 4 mSec.
4. Quickly add 800 µL of YEB medium to the cuvette.
5. Incubate the cells at 28°C for 1 to 2 hours.
6. Spread 200 µL of cell suspension onto a YEB agar plate containing the appropriate antibiotics. For *R. rhizogenes* A13, use rifampicin and streptomycin for selection and kanamycin for the pRI909 binary plasmid.
7. Incubate the plate at 28°C for 2 to 3 days. You should obtain *R. rhizogenes* colonies harboring foreign genes.

This protocol is based on the previous report (Wen-Jun & Forde, 1989).

Transformation of Daughter Plants from Strawberry Stolons

Materials

1. *Rhizobium rhizogenes* A13 strain (MAFF-210266)(Chen et al., 1995) harboring foreign genes, ideally including a fluorescent marker to distinguish whether the emerged hairy roots are transgenic.
2. Stolons from Strawberries (*Fragaria vesca*).
3. YEB or LB medium 0.9% agar plates containing appropriate antibiotics.
4. 18G x 11/2” (ø1.20 x 38 mm) syringe (TERUMO, Japan)
5. Pots filled with vermiculite (W78 mm x D78 mm x H74 mm).

Methods

1. Streak *R. rhizogenes* from glycerol stock on the surface of YEB or LB plates containing the appropriate antibiotics and incubate at 28ºC for 2-3 days.
2. Harvest stolons with 2 to 4 cm-sized daughter plants from strawberry plants.
3. Remove stolons from the crowns of daughter plants by using a scalpel.
4. Place daughter plants on *R. rhizogenes* colonies grown on the surface of YEB or LB plates.
5. Puncture the crowns of the daughter plants with a syringe on the colonies on a YEB or LB plate to infect them.
6. Transplant the infected plants into pots filled with vermiculite, cover them with a wrap to prevent drying out, and grow them for 1 week at 25ºC under 16h light /8 h dark cycles. We transplanted 5 infected plants per pot.
7. One week after infection, remove the wrap.
8. Grow the plants in vermiculite for 1-2 months until adventitious roots emerge.
9. Observe the fluorescence and phenotypes of the adventitious roots, and use the roots with fluorescence for further experiments.

Hairy-root Transformation of Strawberry Seedlings

Materials

1. Strawberry seedlings
2. *Rhizobium rhizogenes* harboring foreign genes.
3. YEB or LB medium 0.9% agar plates containing appropriate antibiotics.
4. Sterile water.
5. 15 mL sterile Falcon tubes.
6. Sterile petri dishes (ø9 cm).
7. Sterile scalpel.
8. 1/2 Gamborg's B5 medium 0.9% agar plates (Gamborg et al., 1968)(FUJIFILM-WAKO, Japan).
9. 1/2 Gamborg's B5 medium 0.9% agar plates containing 12.5 μg l^{-1} of meropenem.
10. Sterile square dishes (9 x 13 cm)

Methods

1. Use seedlings aseptically grown on 1/2 Gamborg's B5 medium 0.9% agar plates.
2. After seedling germination, wrap the plates with an aluminum foil for hypocotyl etiolation for 3 days.
3. Streak *R. rhizogenes* from glycerol stock onto the surface of YEB or LB plates containing the appropriate antibiotics and incubated at 28ºC for 2-3 days.
4. Pick several colonies of *R. rhizogenes* and dilute them in 10 mL sterile water in a 15 mL of sterile Falcon tube until OD_{600} = 0.1.
5. Spread the *R. rhizogenes* bacteria solution on an empty sterile petri dish (ø9 cm).
6. Cut the etiolated seedlings at the bottom end of their hypocotyls to remove roots by using a sterile scalpel.
7. Soak the seedlings in the bacterial solution and co-incubate at room temperature for 15 minutes.
8. Transfer the infected seedlings to 1/2 B5 medium 0.9% agar plates without antibiotics (ø9 cm).
9. Seal the plates with surgical tapes.
10. Co-incubate the seedlings with *R. rhizogenes* on the plates at 25ºC for 3-4 days in the dark.
11. After 3-4 days of incubation with *R. rhizogenes*, transfer the seedlings to solid 1/2 B5 medium 0.9% agar plates containing 12.5 μg l^{-1} of meropenem in square dishes (9 x 13 cm). Usually, 20-24 seedlings are transferred in two rows on a 1/2 B5 plate.

12. Seal the plates with surgical tape at the top and vinyl tape on the other sides. Place the sealed plates vertically, and cultivate the seedlings at 25ºC under 16h light/8 h dark cycles for 2 to 4 weeks.
13. The emerging hairy roots with fluorescence are transgenic and suitable for further experiments.

This protocol is based on previous reports (Díaz et al., 2005;Yan et al., ;2023).

References

Chen, L.-H., Hata, T., Yamakawa, Y., & Suzuki, Y. (1995). The Effects of Preservation Temperatures and Periods on Hairy Roots Inducing Ability of *Agrobacterium rhizogenes*. *Plant Tissue Culture Letters*, *12*(1), 94–96. https://doi.org/10.5511/plantbiotechnology1984.12.94

Díaz, C. L., Gronlund, M., Schlaman, H. R. M., & Spaink, H. P. (2005). Induction of hairy roots for symbiotic gene expression studies. In *Lotus Japonicus Handbook* (pp. 261–277). https://doi.org/10.1007/1-4020-3735-X_26

Gamborg, O. L., Miller, R. A., & Ojima, K. (1968). Nutrient requirements of suspension cultures of soybean root cells. *Experimental Cell Research*, *50*(1), 151–158. https://doi.org/10.1016/0014-4827(68)90403-5

Matsui, T., Sawada, K., Takita, E., & Kato, K. (2014). The longer version of *Arabidopsis thaliana* heat shock protein 18.2 gene terminator contributes to higher expression of stably integrated transgenes in cultured tobacco cells. In *Plant Biotechnology* (Vol. 31, Issue 2, pp. 191–194). https://doi.org/10.5511/plantbiotechnology.14.0117b

Otake, K., Kugou, K., Robertlee, J., Ohzeki, J.-I., Okazaki, K., Hanano, S., Takahashi, S., Shibata, D., & Masumoto, H. (2023). *De novo* induction of a DNA–histone H3K9 methylation loop on synthetic human repetitive DNA in cultured tobacco cells. *Plant Journal*, *114*(3), 668–682. https://doi.org/10.1111/tpj.16164

Wen-Jun, S., & Forde, B. G. (1989). Efficient transformation of Agrobacterium spp. by high voltage electroporation. *Nucleic Acids Research*, *17*(20), 8385. https://doi.org/10.1093/nar/17.20.8385

Yamasaki, S., Sanada, Y., Imase, R., Matsuura, H., Ueno, D., Demura, T., & Kato, K. (2018). *Arabidopsis thaliana* cold-regulated 47 gene 5′-untranslated region enables stable high-level expression of transgenes. *Journal of Bioscience and Bioengineering*, *125*(1), 124–130. https://doi.org/10.1016/j.jbiosc.2017.08.007

Yan, H., Ma, D., Yi, P., Sun, G., Chen, X., Yi, Y., & Huang, X. (2023). Highly efficient *Agrobacterium rhizogenes*-mediated transformation for functional analysis in woodland strawberry. *Plant Methods*, *19*(1), 99. https://doi.org/10.1186/s13007-023-01078-y

The number of transformed hairy roots in each plant

In the plants obtained through transformation, the number of transgenic hairy roots exhibiting fluorescence ranged from one to several. In our hairy-root transformation experiments with daughter plants, plants with multiple transformed hairy roots were frequently observed (Supplemental Figure S1). The hairy roots that emerged from the transformation process underwent active lateral root branching, making it difficult to accurately count the number of transformed hairy roots, distinguish between transformed roots and lateral roots that emerged after transformation, and assess the transformation frequency based on the number of roots. To increase the number of transformed hairy roots per plant, trimming the non-transformed roots with scissors and performing the transformation again might be beneficial. For this purpose, hydroponic cultivation using Falcon tubes or small bottles might be more suitable than vermiculite.

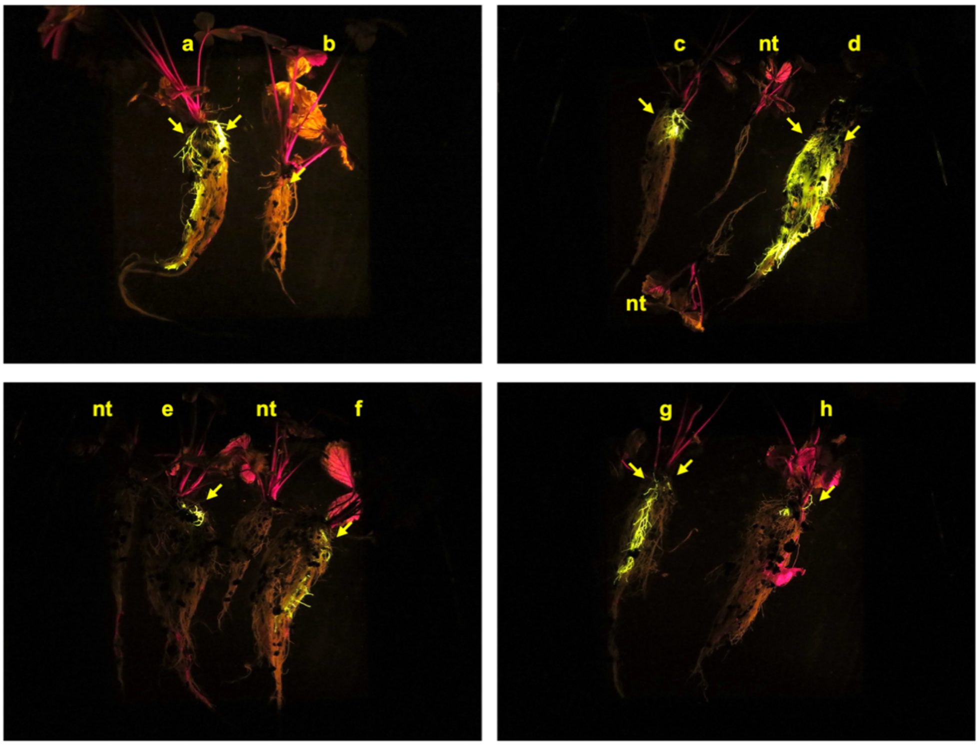

Supplemental Figure S1. The number of transformed hairy roots in each plant. Plants obtained through the transformation experiment are represented. (**a** to **h**) The plants exhibited both transformed and non-transformed hairy roots. Arrows indicate transformed hairy roots with fluorescence. (**nt**) indicates plants with no transformed hairy roots.
